# Relationship between physical activities and mental health in older people: a bibliometric analysis

**DOI:** 10.3389/fpsyt.2024.1424745

**Published:** 2024-10-21

**Authors:** Yuesen Zhang, Mei Zhou, Zhihua Yin, Wenzhen Zhuang, Yufeng Wang

**Affiliations:** ^1^ College of Physical Education and Health, East China Normal University, Shanghai, China; ^2^ Department of General Education, Shanghai Urban Construction Vocational College, Shanghai, China

**Keywords:** older people, physical activity, mental health, sports intervention, CiteSpace

## Abstract

**Objective:**

To summarize the general situation and focal points of research on the physical activity and mental health of older people over the past 15 years and provide references for future research.

**Methods:**

Literature published between January 1, 2009, and December 31, 2023, was retrieved from the Web of Science core database. A bibliometric visualization analysis of countries/regions, institutions, authors, keywords, and references was conducted using CiteSpace6.1.R6.

**Results:**

A total of 4,329 articles were included, and the annual number of articles published over the past 15 years showed an upward trend. The articles were primarily from 65 countries/regions and 626 institutions. The most represented country and institution were the USA and the University of Pittsburgh, respectively. Among the authors identified, Schuch and Callow were the most influential. The research focuses on four areas: the psychological effects of physical activity in older people; physical activity intervention approaches to the mental health of older people; physical activity and mental health assessment questionnaires; and the impact of physical activity on multidimensional aging. Research frontiers involve emerging topics such as the assessment and intervention of mental health in older people and the relationship between their physical activity and cognitive function.

**Conclusion:**

This study conducted a comprehensive, objective, and visual analysis of publications and revealed the status of relevant studies, trending topics, and trends concerning the physical activity and mental health of older people from 2009 to 2023. We hope that this work will help researchers identify new perspectives on potential collaborators, important topics, and research frontiers.

## 1 Introduction

Aging is a chronic, inevitable process that affects the human life cycle, often accompanied by conditions such as cardiovascular disease, hypertension, diabetes, cancer, and mental illness (1). This natural aspect of existence leads to a gradual decline in tissue and organ function, increasing older individuals’ susceptibility to diseases and mortality. Additionally, aging affects the physical, psychological, and pathological conditions of older adults, as well as their social status (2).

Population aging is a significant global trend, driven by substantial improvements in health and survival rates alongside declining fertility rates (3). According to the World Health Organization, “People worldwide are living longer. Today, most people can expect to live into their sixties and beyond. Every country in the world is experiencing growth in both the size and the proportion of older persons.” By 2025, it is projected that individuals aged 60 years and older will constitute 22 percent of the global population (4).

Older adults are particularly vulnerable to age-related illnesses, which has profound implications for individuals and society. Mental health issues are a major concern, with over 20% of individuals aged 60 and older experiencing mental and neurological disorders. These disorders account for 6.6% of total disability within this age group, measured in disability-adjusted life years (5).

Physical activity, defined as body movement produced by skeletal muscle contraction requiring energy expenditure, effectively mitigates the deterioration of physical functions in older adults and promotes healthy aging (6). Research indicates that increased physical activity in later life can reduce mental health disparities associated with educational attainment (7).

Given the urgency to enhance research on physical activity and mental health among older adults, this study employed CiteSpace 6.1.R6 to conduct a comprehensive bibliometric analysis of research on this topic from 2009 to 2023. The aim was to provide new and future scholars with insights into the current state of research, key topics, and emerging trends related to physical activity and mental health in older populations from a global perspective.

## 2 Materials and methods

Scientific knowledge mapping is a visual representation that illustrates the evolutionary processes and relationships between scientific knowledge (8). By employing data analysis, map drawing, and other techniques, researchers can visually represent the intricate data on research institutions, scientific documents, subject structures, and scientific research frontiers using two- or three-dimensional graphics. This visualization aids in analyzing data at the macro, meso, and micro levels, enabling researchers to identify subject focal points, track academic progress, and avoid research blind spots, ultimately facilitating rapid advancement toward the research frontier. After generating the knowledge graph using the CiteSpace software, the focus was mainly on interpreting high-frequency nodes, clustered knowledge groups, and highly mediated centrality nodes.

### 2.1 Source and retrieval

The Web of Science Core Collection was selected as the data source for this study, and an “advanced search” method was adopted. The search formula was as follows: TS=(older adult OR elderly) AND TS=(physical activity and mental health OR exercise and mental health OR sports and mental health). Given the limited number of articles published before 2009, this study focused on English-language studies published between 2009 and 2023 to ensure a systematic and intuitive analysis.

### 2.2 Inclusion and exclusion criteria

This analysis focused solely on studies involving older people aged 60 years and above. The document types considered were limited to articles, review articles, proceeding papers, early access articles, editorial materials, meeting abstracts, book chapters, retracted publications, corrections, and letters.

### 2.3 Screening method

Two reviewers independently screened the articles by evaluating their titles and abstracts according to the predefined inclusion criteria.

**Figure 1:**
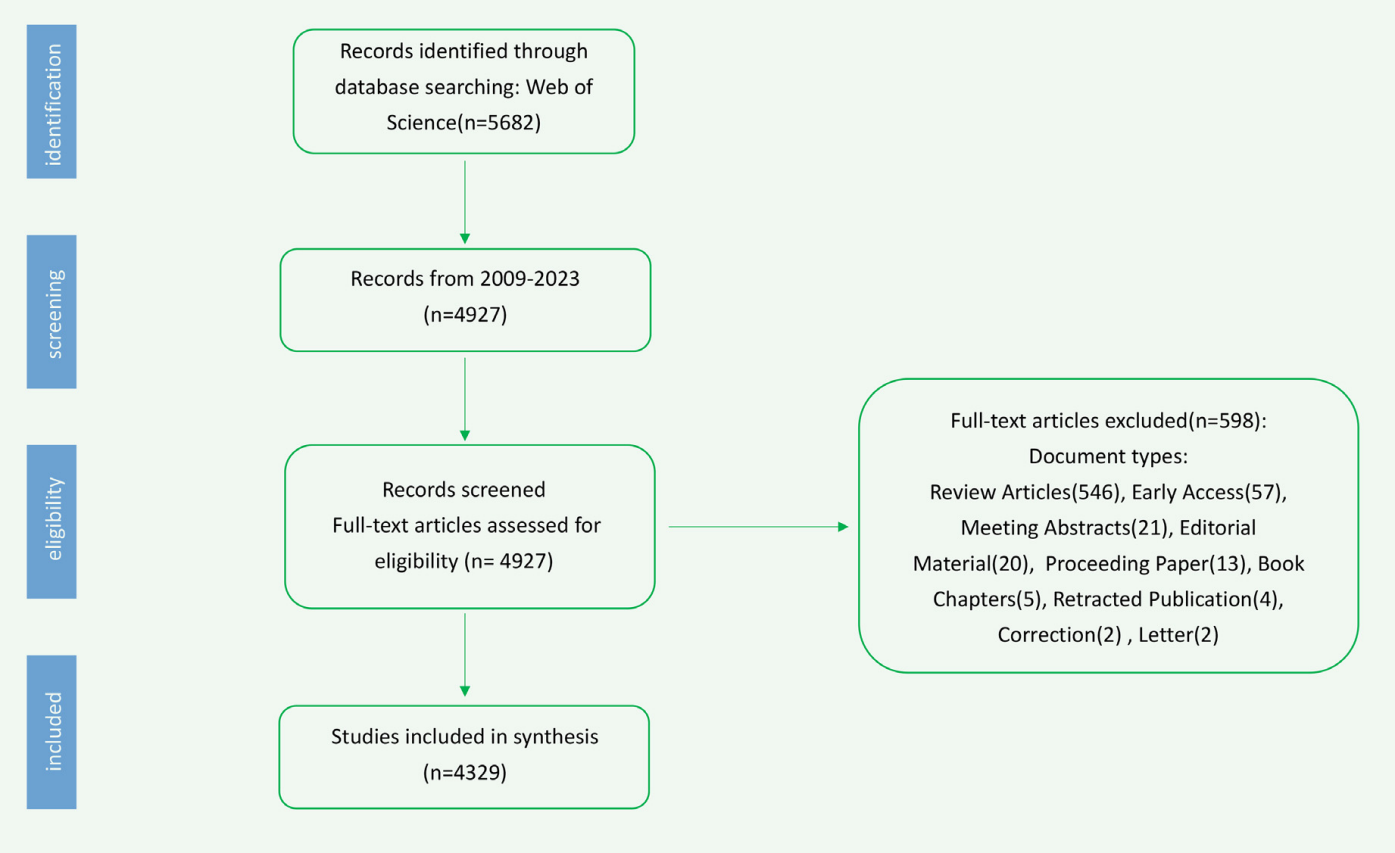
Flow chart of physical activity and mental health in older adult studies inclusion.

A total of 5,682 relevant studies were identified from the Web of Science Core Collection. Following the screening process based on the specified criteria, 4,329 studies were ultimately included (**Figure 1**).

### 2.4 Data acquisition and analytical tools

This study used CiteSpace6.1.R6 for the analysis by combining the literature metrology and visual analysis methods. The relevant documents were manually screened and exported in pure text format with the name “download_xx.txt”.

The effectiveness of CiteSpace mapping is generally assessed using Q- and S-values. The Q-value indicates the clustering module value, and Q>0.3 indicates that the network association structure of the graph is significant. The S-value indicates the average profile value of clustering, and S>0.5 is considered reasonable for the clustering results; when the value reaches 0.7, it indicates that the clustering results are highly reliable. Using Top-N selection as a threshold selection scheme for CiteSpace analysis makes mapping more rational by setting the value of the Top N. The Pathfinder feature simplifies the network and highlights the focus of the map, making it ideal for cropping purposes.

## 3 Results

### 3.1 Annual publication trends

**Figure 2:**
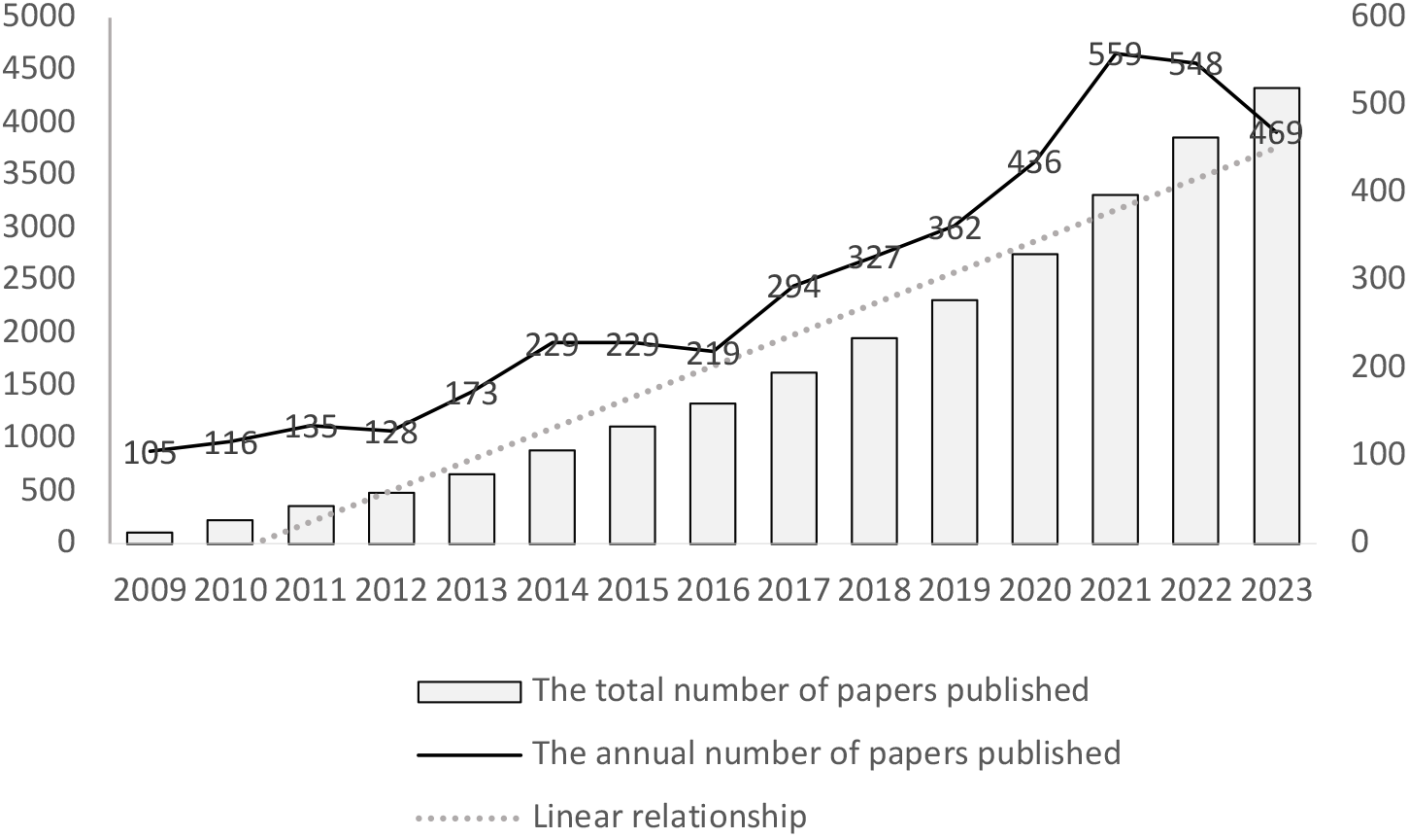
The annual and total number of papers published and the model fitting curve of the time trend of relevant publications.

The annual and total numbers of papers published are critical indicators of academic activity in a field. Analyzing the statistical data of articles related to the correlation between physical activity and mental health in older people can offer valuable insights into the growth rate and potential future directions of research in this area. A total of 4,329 publications were reviewed, and since 2014, the number of articles published each year has exceeded 200. Academic interest in this topic has been steadily increasing, as evidenced by the linear growth in the number of published articles (**Figure 2**). This indicates that the research results and academic exchanges in this field are deepening.

### 3.2 Analysis by country/region

In the country (region) visualization map, the number of nodes was N=65, the number of connections was E=356 (density =0.1712), and the most prominent subnetwork member (largest CC) was 62, accounting for 95% of the total number of nodes. The top 10 countries or regions publishing articles were the United States (1,298 articles), China (628 articles), the United Kingdom (385 articles), Australia (363 articles), Canada (320 articles), Spain (283 articles), Japan (261 articles), Brazil (209 articles), the Netherlands (191 articles), and South Korea (189 articles).

Studies have shown that the global population around the world is aging rapidly. The number of people over 65 years of age is expected to soon exceed the number of children under five years of age (9). However, most research on population aging has concentrated on developed countries (10). This difference may be attributed to the following factors:

#### 3.2.1 Economic support

Developed countries tend to have more robust capital to invest in research on the physical activity and mental health of older adults. These funds can be used to establish research teams, purchase scientific research equipment, and conduct data analyses. In contrast, developing countries require more funds to catch up.

#### 3.2.2 The problem of social aging

Developed countries tend to become aging societies earlier than developing countries. Socioeconomic, medical, health, family structure, and other issues caused by an aging population have become more prominent. Therefore, developed countries will invest more resources in relevant research.

#### 3.2.3 Health awareness and policy orientations

Developed countries usually pay more attention to the health of their citizens, and governments will introduce corresponding policies to promote citizens’ healthy behaviors.

#### 3.2.4 International cooperation

Developed countries usually have more frequent international cooperation and exchanges, which allow them to share research resources, data, and experience, thus promoting the rapid development of research.

### 3.3 Analysis by institution

**Figure 3:**
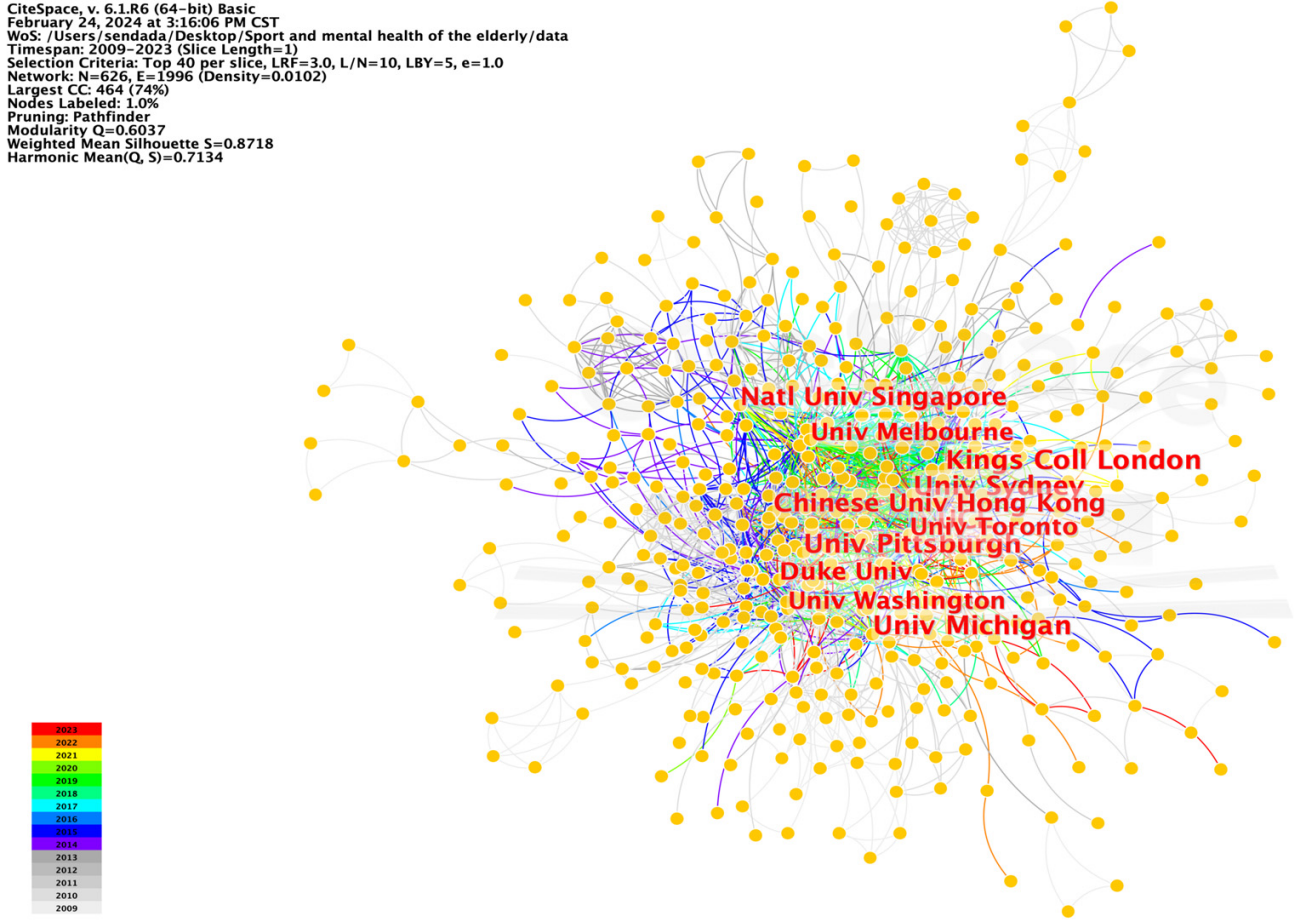
Network mapping of the distribution of research organizations on sport and mental health of older adult people.

As shown in the institutional visualization map, 626 institutions have contributed to the literature on physical activity and mental health in older people. Scientific research institutions comprise comprehensive universities, including 626 nodes and 1,996 connections. The network density was 0.0102, Q-value was 0.6037, and S-value was 0.8718. Clustering results were highly reliable (**Figure 3**).

The institution with the most significant number of publications is the University of Pittsburgh, which published 72 articles. The second-ranked institution is King College London, which published 62 articles, followed by the University of Michigan (61 articles), University of Sydney (61 articles), University College London (61 articles), Chinese University of Hong Kong (53 articles), National University of Singapore (52 articles), University of Melbourne (49 articles), Duke University (46 articles), and University of Toronto (46 articles).

Bursting indicates large fluctuations in the data over a short period. Burst functions can locate scientific research institutions with significant growth in results. The emergence map of research institutions shows the distribution of the citation intensity of the 25 universities and research institutes. The University of Oslo and Karolinska Institute began research on the physical activity and mental health of older adults as early as 2009. The University of Barcelona and Katholieke Universiteit Leuven had the highest citation intensities, at 10.89 and 10.51, respectively.

### 3.4 Literature analysis of key nodes

Key node literature refers to documents that introduce significant theoretical advancements in a particular field, which are often considered pivotal moments in knowledge development. Identifying these knowledge turning points involves identifying the key nodes that are prominent within the visualization network. When two documents are cited in a third document, they form a co-citation relationship. Analysis of these relationships through a collection of literature spatial data is known as co-citation analysis. The goal of this analysis is to identify key nodes in the literature in the evolution of a particular knowledge area.

**Figure 4:**
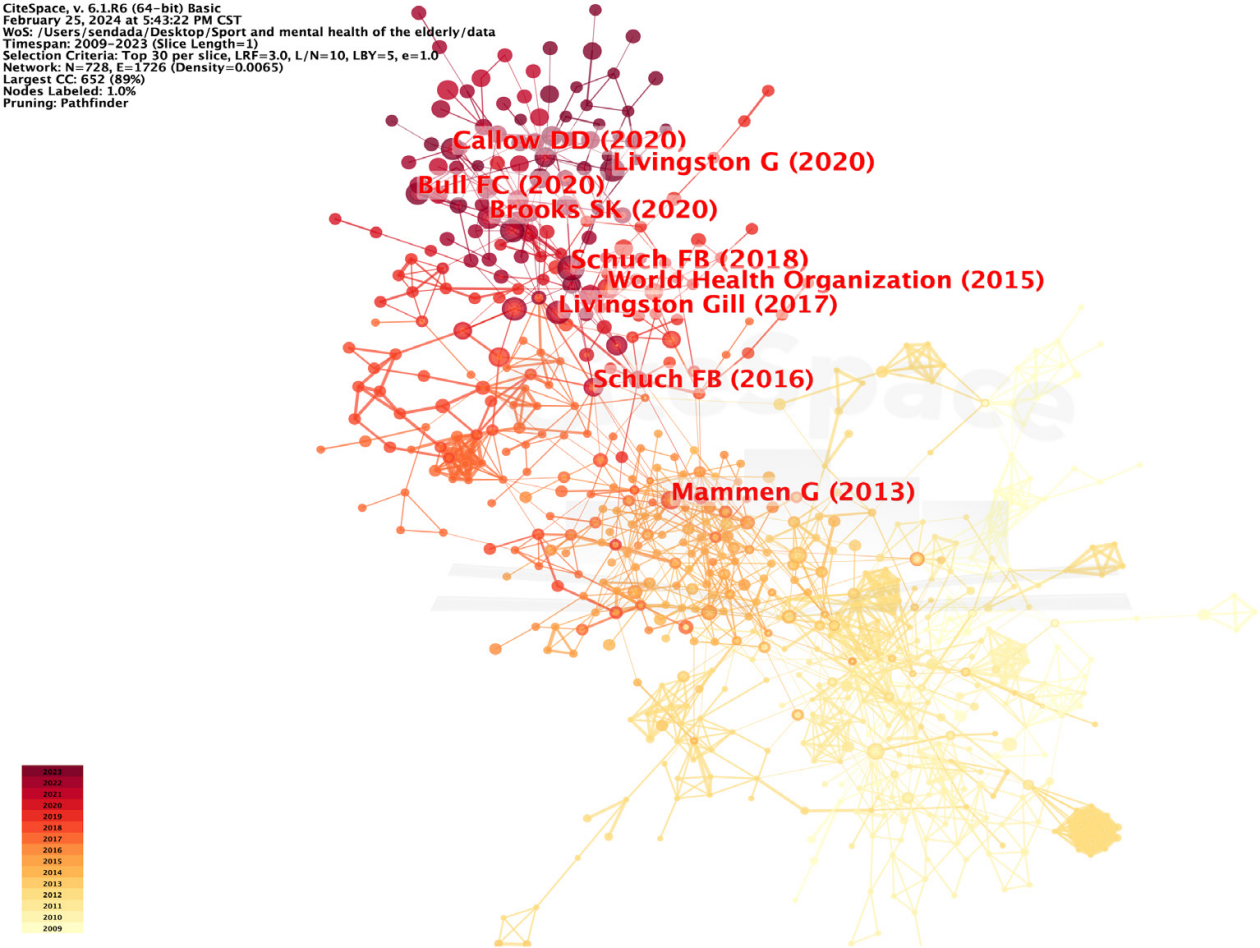
Co-citation mapping of literature on physical activity and mental health research on older adult people.

This study used the literature co-citation analysis function of CiteSpace6.1.R6 software to obtain key node documents in the evolution of research on the physical activity and mental health of older people. The node type parameter was set to Reference, the number of years per slice was set to 1, and the threshold value was Top N=30. Visual analysis of the co-cited references was performed in CiteSpace, and a map with 728 nodes and 1,726 connections was generated. Findings indicated Density=0.0065, Modularity Q=0.8129, and the clustering effect evaluation parameter Mean Silhouette S=0.9065; the clustering results had high confidence (**Figure 4**).

The literature co-citation map helps researchers analyze the evolution of a research topic through key nodes, clusters, and map colors. Centrality and high citation frequency were the key nodes in the network for literature co-citation mapping. Co-citation mapping shows that the most cited article is “Physical Activity and Incident Depression: A Meta-Analysis of Prospective Cohort Studies,” published by Felipe B. Schuch in 2018 and cited 1,364 times. This review highlighted the impact of physical activity on both physiological indicators, such as hippocampal volume and neurogenesis marker levels, and psychological factors, including self-esteem and the enhancement of embodied cognitive abilities. Consequently, individuals with higher physical activity levels are less likely to develop depression (11). The second most frequently cited article was Daniel D. Callow’s “The Mental Health Benefits of Physical Activity in Older Adults Survive the COVID-19 Pandemic,” published in 2020 and cited 187 times. This study examined 1,046 Americans aged 50 years and older, revealing that approximately 45% had encountered anxiety or depression in the course of the COVID-19 pandemic. It delved into the mental health advantages of physical activity for older adults, thus navigating the challenges of the pandemic. The research also indicated that older adults engaging in physical activity exhibited reduced levels of depression and anxiety symptoms compared to their less active counterparts. These findings imply that the mental health benefits of physical activity can be amplified by higher levels of intensity (12).

### 3.5 Centrality analysis of literature

**Figure 5:**
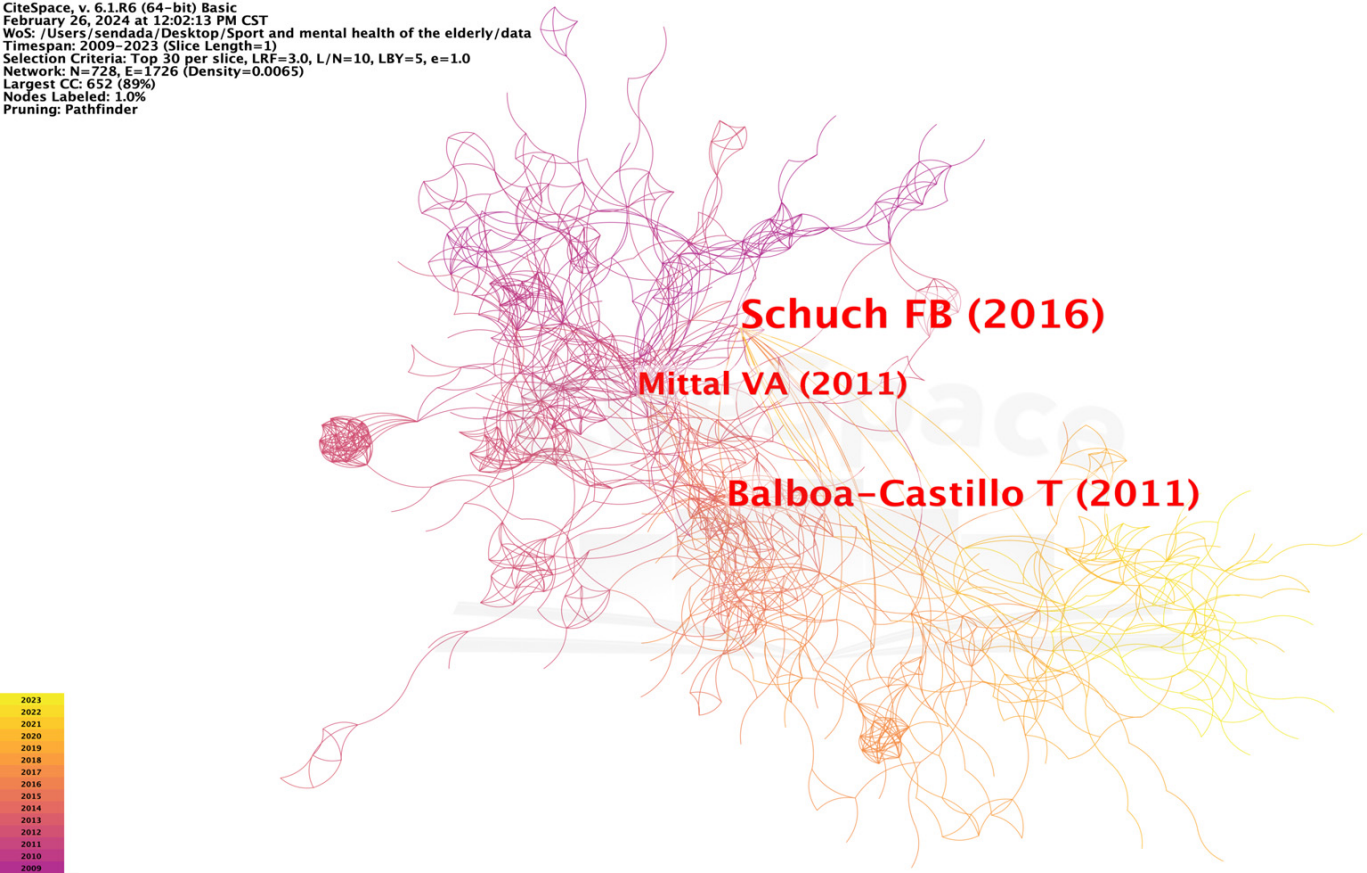
Centrality mapping of research for physical activity and mental health in older adult people.

Centrality is a metric used in CiteSpace to discover and measure the importance of literature; literature with high intermediary centrality is often a key hub connecting two different domains. Upon sorting the literature by mediational centrality (**Figure 5**), Schuch’s “Exercise as a Treatment for Depression: A Meta-Analysis Adjusting for Publication Bias,” published in 2016, ranked first with a mediational centrality of 0.27. This study employed a meta-analysis approach to systematically examine published randomized controlled trials that investigated the effects of exercise on depression. These findings suggest that exercise has a notable therapeutic impact on depression, particularly in individuals with mild to moderate depression (13).

Moreover, the article “Longitudinal Association of Physical Activity and Sedentary Behavior during Leisure Time with Health-Related Quality of Life in Community-Dwelling Older Adults,” published by Balboa-Castillo in 2011, ranked second with a centrality of 0.25. This study highlighted the safety and benefits of leisure physical activities such as walking for older people. This also suggests that substituting extended periods of sitting with light physical activity can enhance health-related quality of life (14). Analysis of the key nodes in the literature shows that research on physical activity and mental health in older adults has centered on the protective effect of physical activity on psychological depression or anxiety. This study aimed to enhance the mental health of older adults and explore the impact of various types of exercise on their physical well-being.

### 3.6 Analysis of keywords

The co-occurrence analysis method focuses on the statistical analysis of the number of times a group of words co-occurs in the same piece of literature, with the main focus on clustering and data visualization methods. The keyword co-occurrence map included 134 nodes and 594 connecting lines.

Word frequency analysis is a bibliometric method that uses the frequency of keywords or subject words to reveal or express the core content of documents in a certain research field to determine research focal points and development trends. Nine keywords were used more than 550 times (**Table 1**).

**Table 1:** Keyword frequency for research on the physical activity and mental health of older people (count≥550).

Keywords	Count	Centrality
The elderly	1,566	0.17
Physical activity	1,544	0.18
Mental health	1,061	0.14
Health	990	0.16
Quality of life	791	0.11
Exercise	612	0.02
Adult	583	0.1
Risk	569	0.07
Association	550	0.13

In the Timeline View, documents within the same cluster are displayed on the same horizontal line. This visualization provides a clear representation of the volume of literature within each cluster, with a greater number of clusters indicating more significant research areas. Furthermore, the Timeline View allows for the observation of the period of literature within each category, as well as the evolution of specific research clusters from emergence to peak and eventual decline.

The focus of cutting-edge research has shifted over time. Between 2009 and 2015, there was a significant growth in research on the impact of physical activity on the mental health of older people. The primary emphasis was on promoting physical health and preventing disease in the older population. This included evaluating their physical capabilities and overall health as well as exploring ways to enhance their quality of life through physical activity and ongoing support. Research on the relationship between physical activity and mental health in older adults advanced significantly between 2015 and 2019. Previous studies systematically investigated the effects of various exercise intensities on cognitive impairment in older individuals using randomized controlled trials and questionnaire surveys. From 2019 to 2023, the focus of research shifted toward examining the impact of mental health on the overall quality of life. Researchers are primarily investigating how older adults can improve their mental well-being through interventions involving physical activity.

**Figure 6:**
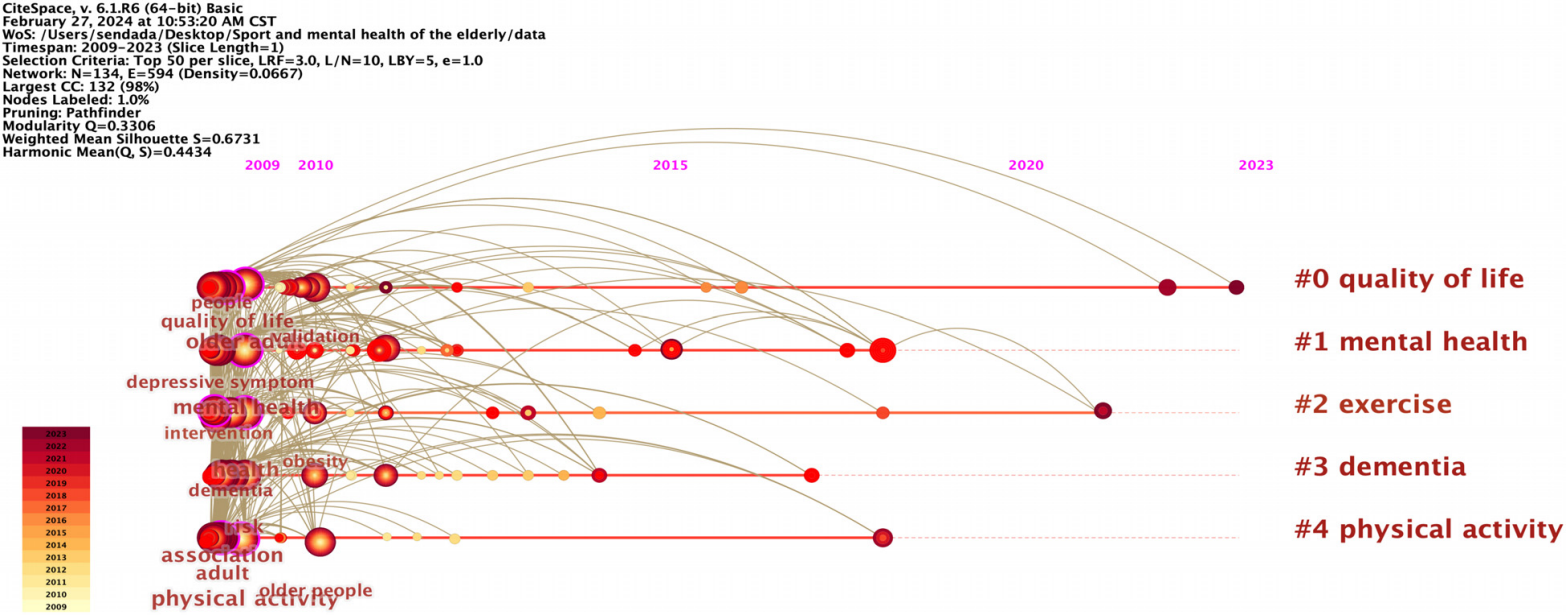
Timeline view map of keywords for physical activity and mental health research in older adult people.

Setting the Layout and Timeline in the Control Panel, in turn, can obtain a Timeline View map, Modularity Q=0.3306, and Mean Silhouette S=0.6731; the clustering result is reasonable (**Figure 6**). The cluster of “#0 quality of life” is prevalent in the field, with recent research emphasizing “social participation” and “life satisfaction.” The “#1 mental health” cluster delved into the symptoms of anxiety, depression, and loneliness commonly observed in older adults. Thirty-eight articles were published based on the study on social isolation behavior in “#2 workouts.” Since 2015, the emergence of keywords such as “aerobic exercise,” “psychotherapy,” “cognitive bias,” and “sedentary behavior” has provided new research directions.

Analysis was conducted to identify research focal points and structural divisions, resulting in four major themes: research on the psychological effects of physical activity on older people, research on intervention methods for physical activity on older people’s mental health, research on physical activity and mental health assessment questionnaires for older people, and the effects of physical activity on multidimensional aging.

## 4 Discussion

### 4.1 Research on the psychological effects of physical activity on older people

Although older people play a crucial role in many societies, they often face greater vulnerabilities (15). Similar to other age groups, the well-being of older people is influenced by various factors, such as traits, physical and mental health, psychological well-being, social connections, and daily activities. The mental health status of older people is a significant concern. It is intricately linked to their quality of life and overall level of happiness (16, 17). The promotion of mental health is a crucial aspect of physical activity. Studies have demonstrated that engaging in physical activity that involves significant calorie expenditure can be an effective method for addressing mental health disorders (18). This study synthesized a substantial number of randomized controlled trials to investigate the protective effects of physical activity on the mental health of older adults. These findings strongly support the positive impact of physical activity on the mental well-being of older adults.

#### 4.1.1 Effects of physical activity on depression in older people

Depression is a mental disorder characterized by feelings of sadness, reduced interest in daily activities, concentration challenges, and memory issues (19). Depression is a prevalent mental health condition among older adults, with the highest occurrence observed between the ages of 60 and 70 years. Women are affected by depression at a rate approximately twice as much as men (20). Physical exercise has attracted much attention over the past decade as a less expensive treatment for depression with fewer side effects (21). Schuch et al. found that 67.8% (95% confidence interval [CI]: 52.1–80.3) of patients with depression did not adhere to the recommendation of 150 minutes of moderate or vigorous physical activity per week (22). Experiments exploring the correlation between the frequency, intensity, and duration of muscle-strengthening activities and mental health have demonstrated that engaging in muscle-strengthening activities at least once per week can help prevent depression. Furthermore, it has been observed that the more frequent the physical exercise, the fewer the symptoms of anxiety and depression (23). Other studies have found that physical activity of any intensity, including low levels of physical activity, can protect against depression (24, 25). Previous studies have shown that physical activity can be effective in treating depression; however, further research is needed to determine its specific impact on older adults. López-Torres’ randomized clinical trial, focusing on patients aged 65 and above with clinically significant depression in a primary care setting, demonstrated that physical activity can revolutionize the treatment of depression in clinical practice (26). This alternative approach allows older individuals to potentially decrease their reliance on medication, while avoiding the side effects associated with traditional drug therapies. These findings have significant theoretical implications for the prevention and management of depression in older adults (27).

Research on the impact of physical exercise on depression in older adults during the COVID-19 lockdown revealed a negative correlation between physical exercise and depressive symptoms. Mental toughness plays a mediating role in the effect of physical exercise on depression in older adults and can serve as a negative predictor of depression (28). Hofman et al. conducted a study on 1,943 older individuals with an average age of 71 years (29). The results showed that depressive symptom scores were significantly lower by -0.55 (95% CI: -1.04, -0.06) points in older people when sleep was replaced with 30 minutes of moderate- to high-intensity physical activity. Similarly, depressive symptom scores were lower by -0.59 (95% CI: -1.06 to -0.12) points when replacing sedentary behavior with 30 minutes of moderate- to high-intensity physical activity. This suggests that maintaining moderate- to high-intensity physical activity is important for older adults. Research on Australian individuals over the age of 65 years during the COVID-19 pandemic indicated that engaging in group physical activities could decrease symptoms of depression and other mood disorders while fostering a sense of belonging and social support (30). It is recommended that older people enhance their knowledge of disease prevention and control during pandemics and boost their immune systems through structured physical activities.

The effect of physical activity on depression in older individuals is also influenced by gender stratification. Research conducted in a southern Brazilian community found that higher levels of physical activity were associated with lower rates of depressive symptoms, particularly among men. Physically active men had a 68% lower risk of depressive symptoms compared to the inactive group (95% CI: 0.12-0.86), whereas physically active women had a 24% lower risk (95% CI: 0.39-1.46) (31).

#### 4.1.2 Effect of physical activity on cognitive function in older people

Cognitive function is the process of acquiring or applying knowledge and external information. It is a method of information processing and is the most basic psychological process of human beings. The decline in cognitive function varies from mild cognitive impairment to dementia, resulting in significant personal, social, and economic burdens (32). Global aging is expected to increase the number of cases. The prevalence of dementia increased from 20.2 million in 1990 to 43.8 million in 2016 (33).

Exercise and regular physical activity are effective in promoting cognitive improvement and brain function. Analysis of magnetic resonance imaging images of 165 older individuals without dementia revealed a significant correlation between health, spatial memory performance, and left and right hippocampal volumes. Higher levels of aerobic exercise have been linked to an increased hippocampal volume in older individuals, suggesting that physical activity could lower the risk of dementia (34). Recently, numerous exercise intervention experiments have been conducted to examine the causal relationship between exercise and cognitive function. Physical activity patterns such as slow gait are significantly associated with an elevated risk of cognitive decline and dementia (35). An experiment involving 110 women aged 60–91 years revealed a significant relationship between engaging in physical exercise and psychological functions, particularly cognition. Maintaining a level of physical activity equivalent to approximately 5,000 steps per day is a crucial factor in safeguarding older people against cognitive decline and dementia (36). A survey conducted in Taiwan among individuals aged 65 years and older (n=2,724, male: 1,348) revealed that the prevalence of cognitive impairment in this demographic group was 24.4%. This study found a significant association between leisure-time physical activity and cognitive impairment (p=0.01), indicating that older adults who did not engage in leisure-time physical activity were at a higher risk of developing cognitive impairment (37).

#### 4.1.3 Effect of physical activity on life satisfaction in older people

Life satisfaction refers to an individual’s cognitive evaluation of their own life and is a crucial component in assessing mental well-being (38, 39). With the ongoing aging of the society and the rise in average life expectancy (40), understanding the life satisfaction of older people is becoming increasingly important. Recent research has revealed a growing trend in exploring the relationship between physical activity and life satisfaction among older adults, focusing on deepening and expanding horizontally. Toros et al. conducted a comprehensive study involving 215 men over 65 years of age and revealed a significant difference in life satisfaction levels between older men who engaged in regular exercise and those who did not (41). Maher et al. demonstrated a positive correlation between regular physical activity and life satisfaction in middle-aged and older individuals. Engaging in regular physical activity has been shown to enhance life satisfaction in older people (42). Sharifi et al. discovered a notable positive relationship between physical activity and psychological adjustment among retired individuals. Their study revealed a strong correlation between high levels of physical activity and improvements in life satisfaction, quality of life, and psychological well-being (43). Kim et al. conducted regression analyses on health and retirement data, revealing that recreational physical activity significantly contributes to enhancing the quality of life and maintaining mental health in older people with mild cognitive impairment (44). Engaging in recreational physical activities not only provides a range of enjoyable experiences that increase life satisfaction but also effectively reduces psychological stress related to cognitive impairment and enhances mental well-being.

Several studies have demonstrated other aspects of the role of regular physical activity in life satisfaction (45). A meta-analysis of 36 datasets found that physical activity had significant effects on individuals aged over 65 years, particularly in terms of improving health and quality of life. Aerobic exercise is particularly beneficial in promoting mental health (46). Studies conducted with community-dwelling seniors in Canada have demonstrated that consistent aerobic exercise and short-term exercise interventions can decrease the likelihood of functional limitations and disability in later life while also mitigating the physical and psychological effects commonly linked to the aging process (47). Empirical evidence suggests that regular physical activity among people aged over 65 years has a positive impact on life satisfaction (48).

Longitudinal studies have employed follow-up surveys to extensively examine the ongoing changes in the lasting influence of physical activity on the life satisfaction of older individuals. In contrast, cross-sectional studies have uncovered various intricate connections between physical activity and life satisfaction in older people by analyzing differences across groups and geographic regions. This multidimensional research approach enhances our understanding of the correlation between physical activity and life satisfaction in the older population.

### 4.2 Research on intervention methods of physical activity on the mental health of older people

Physical activity intervention is a multidisciplinary process that focuses on movement and draws on theories and methods in sociology, medicine, and environmental studies. A study on physical activity for mental health in older people aimed to reduce sedentary behaviors and improve the health status of older people (49). Exercise can be a beneficial alternative to medication for the treatment of depression in older populations. Research findings from both prospective and experimental studies support the effectiveness of aerobic exercise; resistance training; and mind-body exercises such as tai chi, fitness qigong, and yoga (50).

#### 4.2.1 Resistance exercise interventions

Resistance exercises, also known as strength training, encompass various routines that stimulate muscle responses through resistance (51). Marrella et al. conducted semi-structured interviews and discovered notable health benefits associated with resistance exercise. Individuals burdened by heavy loads and engulfed in negative emotions reported that strength training helped alleviate their fragile mental states (52). Following high-intensity (80% maximal load) or low-intensity (20% maximal load) progressive resistance training in 60 older adults aged > 60 years with major or minor depression, Singh et al. observed a direct correlation between decreased depressive symptoms and increased strength (r=0.40, p=0.004). All participants exhibited notable enhancements in sleep outcomes (p<0.001), with the most significant improvements observed in those who underwent high-intensity progressive resistance training (p=0.05) (53). Research has demonstrated that resistance exercise offers numerous physiological and psychological advantages. Intensive resistance training is effective in enhancing mood, reducing anxiety, and increasing insulin-like growth factor-1 serum concentrations, which is a mechanism for mood improvement in older individuals without clinical mood disorders (54).

However, some scholars have argued that there is still a need for more convincing theoretical explanations. An eight-week resistance training intervention was conducted with 62 community-dwelling women with an average age of 68 years. The results indicated that there were no significant improvements in the mental or physical health functions of older participants in the experimental group compared to the control group. The researchers hypothesized that changes in mental health function may occur over an extended period of resistance training, or that other social factors associated with the program may impact the enhancement of mental health in older adults (55).

#### 4.2.2 Aerobic exercise interventions

Aerobic exercise serves as a potent stimulus to enhance mitochondrial mass and improve skeletal muscle function (56). This type of exercise is beneficial for enhancing the immune system and preventing or managing chronic conditions such as cardiovascular disease and diabetes (57). Bennie et al. analyzed sample data from 1.5 million individuals and revealed that engaging in moderate- to high-intensity aerobic physical activity and muscle-strengthening activity can decrease the occurrence of depression (58). An analysis of 47,564 American adults revealed that consistent aerobic exercise has several beneficial effects on sleep quality. These include a decrease in the time taken to fall asleep, reduction in daytime sleepiness, and increase in the duration of deep sleep (59). Fluetsch et al. conducted an analysis using the Behavioral Risk Factor Surveillance System that involved a sample of 441,456 individuals. Their findings suggest that physical activity has the potential to decrease psychological ill-health related to anxiety, depression, and stress in a dose-response manner (60).

#### 4.2.3 Tai chi and fitness qigong interventions

Chinese qigong is a beneficial mind-body exercise regimen for enhancing lower-body physical function and increasing feelings of revitalization following exercise (61). Research findings indicate that tai chi and qigong, as non-pharmacological interventions, are effective in alleviating depressive symptoms, reducing stress, managing anxiety, and improving mood disorders (62). Lavretsky et al. conducted a randomized controlled clinical trial with 112 older people aged 60 years and above with severe depression. This study revealed that supplementing depression medication with tai chi could enhance its effectiveness in this population, leading to improvements in the quality of life and memory (63). For older individuals with chronic health conditions, qigong may be a beneficial form of exercise because of its positive effects on physical and functional abilities and balance, as well as its ability to reduce symptoms of depression and anxiety (64).

### 4.3 Research on physical activity and mental health assessment questionnaires for older people

Hurtig et al. adapted the short-form version of the International Physical Activity Questionnaire (IPAQ) to better suit the unique requirements of individuals aged 65 and above. The revised questionnaire, the IPAQ for the Older adult, has been thoroughly validated (65). Van Holle et al. adapted a long-form version of the IPAQ for older people in a Belgian community. The questionnaire demonstrated moderate validity in measuring weekly minutes of moderate-to-vigorous physical activity and the total time spent on physical activity (66). Forsén conducted a systematic review and evaluation of three self-administered physical activity questionnaires designed for older individuals. The study confirmed the reliability of the following three questionnaires: IPAQ–Chinese version, with an intraclass correlation coefficient of ≥0.81; Women’s Health Initiative—Physical Activity Questionnaire, with an intraclass correlation coefficient of 0.76; and Physical Activity Scale for the Older adult, with a Pearson correlation coefficient of 0.84 (67).

Life satisfaction, self-esteem, and self-efficacy are crucial factors that influence the mental well-being of older adults. Life satisfaction is influenced by disciplines such as psychology and sociology (68). Lower levels of depression and a stronger sense of belonging play distinct roles in influencing self-esteem in the older population (69). Individuals with high self-efficacy are more likely to engage in health-promoting behaviors, maintain social connections, and effectively regulate their emotions (70). In 1985, Diener et al. developed the Satisfaction with Life Scale consisting of five items. Participants were asked to rate each item on a seven-point scale based on their satisfaction levels. This scale is commonly used to assess subjective well-being and ordinary life satisfaction (71). The Coopersmith Self-Esteem Inventory is a self-assessment scale developed by Coopersmith et al. Participants rate each item on a scale, with higher scores reflecting higher self-esteem (72). The Self-Efficacy Scale is a mental health assessment tool developed by Riggs et al. It focuses on measuring confidence in self-efficacy across 10 criteria (73).

Questionnaires are cost-effective methods to assess the level of physical activity in daily life (74). The IPAQ is a commonly utilized tool in these studies. Researchers from diverse regions have adapted and enhanced the questionnaire to suit older populations from various cultural backgrounds, ensuring its reliability and validity (75–79).

### 4.4 Effects of physical activity on multidimensional aging

The global population is aging, as evidenced by epidemiological studies indicating that 11% of individuals are over 60 years of age. Projections suggest that this percentage will double to 22% by the year 2050 (80). Over time, it has evolved into three main directions: “healthy aging,” “active aging,” and “successful aging” within the framework of the “activity perspective” in social gerontology.

The World Health Organization (WHO) defines healthy aging as the process of developing and maintaining functional abilities to promote well-being in older adults (81). Healthy aging has traditionally been assessed using cognitive and functional abilities that serve as proxy markers for brain health (82). The increasing trend of global aging has made healthy aging a crucial social issue in many countries. Studies on physical activity interventions, specifically balance and resistance training, have demonstrated that regular physical activity can enhance physical function, prevent falls and disabilities in daily life, reduce negative physiological changes related to aging, and enhance psychosocial health and well-being in older individuals, ultimately contributing to healthy aging (83). McPhee et al. emphasized the importance of regular physical activity in enhancing both the physical and mental well-being of older people. The transition from low-intensity walking to vigorous activity and resistance exercise has been shown to decrease the likelihood of obesity, falls, cognitive decline, osteoporosis, muscle atrophy, and cardiovascular and metabolic disorders (84).

Active aging was originally defined by the WHO as the process of optimizing opportunities for health, participation, and security to enhance quality of life as people age (85). The term “active” denotes ongoing engagement in social, economic, cultural, spiritual, and civic activities, extending beyond physical and professional activities (86). The Active Ageing Index (AAI) is composed of four domains: (a) employment; (b) social participation; (c) independent, healthy, and safe living; and (d) capacity for active aging and a favorable environment. These domains are further divided into 22 indicators (87). A study conducted in Malaysia investigated the relationship between active aging and mortality risk among a sample of 2,230 individuals aged 60 and above. The results of the Cox regression analysis revealed that active aging was associated with a 2.5% reduction in the risk of mortality among older people after controlling for variables such as sex, marital status, age, race, chronic diseases, and risk factors (88). Cross-country comparisons of active aging levels across different age groups in EU member states using the AAI revealed systematic variations between countries (89). Countries with higher levels of active aging typically have more evenly distributed populations of actively aging individuals.

In recent years, skepticism surrounding AAI has grown. Jensen et al.’s study of active aging, which utilized the AAI, revealed a conceptual framework that lacked coherence and a strong theoretical foundation. Active aging is influenced by both the physical and mental well-being of individuals as well as their social status (90).

The theory of “successful aging” put forth by Rowe and Kahn is prevalent in the American discourse on aging. It comprises three key components: reduced risk of disease, enhanced physical and cognitive abilities, and active engagement in society (91). Harmell defined a successful aging strategy as a potentially modifiable characteristic or intervention aimed at enhancing the functioning of older people who are aging normally (92). Pruchno et al. emphasized the importance of understanding the concept of “successful aging” from a life cycle perspective. They proposed a two-factor model that incorporated both objective and subjective factors (93). Similarly, Kanning et al. emphasized the importance of physical activity in the context of “successful aging.” Physical activity enables individuals in good physical health to pursue personal goals and directly address their psychological needs (94). Gopinath et al. studied the temporal relationship between physical activity and successful aging (95). They found that high levels of physical activity can increase the probability of “successful aging.”

The combination of declining fertility rates and rising life expectancy is causing the rapid aging of populations worldwide (96). Healthy aging is determined by not only individual success and motivation but also the support and opportunities provided by the society to help older individuals maintain their functionality and continue to engage in activities that they value (97). Active aging is a holistic approach aimed at optimizing individuals’ engagement and quality of life as they grow older (98). Over the past five decades, the concept of successful aging has undergone significant evolution, transitioning from initial theories centered on activity and disengagement to more direct theoretical approaches (99). Physical activity plays a crucial role in achieving these aging objectives, as it greatly contributes to enhancing physical function, promoting mental well-being, and improving the overall quality of life of older people.

## 5 Limitations

Despite conducting a thorough visual analysis of publications on the relationship between physical activity and mental health in older adults, this study was limited in several ways. First, the search for relevant articles was limited to the Web of Science and did not include any other databases. Second, the impact of physical activity interventions on the mental health of older adults may be influenced by factors such as the type, intensity, and duration of the intervention. This study focuses on English-language literature published between 2009 and 2023, which may lead to the exclusion of earlier findings or studies published in non-English languages.

## 6 Conclusion

This study highlighted the significant research findings, key themes, and emerging trends in the relationship between physical activity and mental health among older adults between 2009 and 2023. The number of relevant articles has steadily increased over the past 15 years. Current studies are predominantly conducted in developed nations and universities and research institutions. This study focused on four main areas: first, examining the psychological effects of older individuals engaging in physical activities; second, exploring effective interventions to improve the mental health of seniors through physical activities; third, enhancing the connection between physical activities and the psychology of older people through health assessment questionnaires; and fourth, investigating the multidimensional impact of physical activity on aging. Psychological researchers have focused on studying the effects of physical activity on the mental health of older individuals, specifically examining changes in depression, cognitive function, and life satisfaction. Various intervention methods, including resistance exercise, aerobic exercise, and other forms of exercise, have been used extensively to demonstrate the beneficial effects of physical activity on the mental well-being of older adults.

To accurately evaluate the physical activity levels of older individuals, researchers from various countries have adapted the IPAQ into long and short versions. Additionally, assessment tools, such as the Physical Activity Scale for the Older adult, Satisfaction with Life Scale, and Self-Efficacy Scale, have been developed. Physical activity is essential for promoting the concepts of healthy, active, and successful aging. Analyzing the research progression from 2009 to 2015, the primary focus was on “disease prevention.” Subsequently, between 2015 and 2019, there was a gradual transition towards studying “sport and cognition.” Finally, from 2019 to 2023, the main research emphasis shifted to “sport for mental health.” To meet the health needs of the older population and promote the harmonious development of an aging society, it is essential to promote physical activity methods for older people at the grassroots level.

## Data Availability

The original contributions presented in the study are included in the article/supplementary material. Further inquiries can be directed to the corresponding author.
